# Enteroparasitosis infections among renal transplant recipients in Khartoum state, Sudan 2012–2013

**DOI:** 10.1186/s13104-018-3716-8

**Published:** 2018-08-29

**Authors:** Nouh Saad Mohamed, Emmanuel Edwar Siddig, Mona Ali Mohamed, Basma AbdlMoniem Alzein, Hanaa Hashim Saeed Osman, Emmanuel E. Tanyous, Bahaeldin K. Elamin, Ali Mahmoud Mohammed Edris

**Affiliations:** 1 Department of Parasitology and Medical Entomology, Faculty of Medical Laboratory Sciences, Sinnar University, Sinnar, Sudan; 2 Department of Molecular Biology, National University Research Institute, National University, Khartoum, Sudan; 3 Department of Parasitology and Medical Entomology, Faculty of Medical Laboratory Sciences, Nile College, Khartoum, Sudan; 4 Department of Histopathology and Cytology, Faculty of Medical Laboratory Sciences, University of Khartoum, Khartoum, Sudan; 5 Mycetoma Research Center, University of Khartoum, Khartoum, Sudan; 6 Department of Histopathology and Cytology, Ibn Sina University, Khartoum, Sudan; 7 Department of Histopathology and Cytology, Faculty of Medical Laboratory Sciences, Port Sudan AL-Ahlia College, Port Sudan, Sudan; 8 Department of Biostatistics and Central Supervision Unit, Ministry of Health, Riyadh, Kingdom of Saudi Arabia; 9 Department of Medical Microbiology, Faculty of Medical Laboratory Science, University of Khartoum, Khartoum, Sudan; 10 Department of Basic Sciences, College of Medicine, University of Bisha, Bisha, Kingdom of Saudi Arabia; 11 Department of Histopathology and Cytology, Faculty of Applied Medical Sciences, University of Bisha, Bisha, Kingdom of Saudi Arabia

**Keywords:** Renal transplant, Parasitic infections, Khartoum, Sudan

## Abstract

**Objectives:**

Renal transplantation procedure markedly increased over the past few decades. The risk of harboring parasitic diseases may affect transplant recipients during life expectancy. We aimed in this study to determine the enteroparasitosis frequency among renal transplant recipients in Khartoum state, Sudan. A case–control hospital-based study performed between November 2012 and May 2013, on 300 renal transplant recipients attending Sudanese Kidney Association hospital in Khartoum state, Sudan, along with 300 normal healthy individuals matching the case in age and sex. Stool samples were collected for parasitological studies.

**Results:**

Out of the 300 renal transplant recipients: 242 (80.7%) were males mean age 43 ± 11.28 and 58 (19.3%) were females mean age 41 ± 13.41. Intestinal parasitic infection was observed in 118 participants and the overall frequency was 19.7%; of which 64 were cases (21.3%) and 54 (18.0%) were controls. Eight different species of intestinal parasites were identified; *Entamoeba histolytica/dispar* (7.5%), *Entamoeba coli* (6.5%), *Giardia lambelia* (3.2%), *Cryptosporidium parvum* (1.2%), *Ascaris lumbricoides* (0.6%), *Enterobius vermicularis* (0.3%), (0.2%) for each of *Strongyloides stercoralis* and *Hymenolepis nana*.

## Introduction

Renal transplantation has significantly increased over the past few decades [1]. In 2013, 75,000 kidney transplants were done worldwide in the year 2010 [2]. It is also the only treatment means for devastating stage of renal failure [3]. However, the long-lasting endurance of renal tissues so far remains a baffling and unsolved question as the majority of the renal transplant recipients go through failure within a decade of their transplantation [4].

Parasitic infections occurring in renal transplant recipients are still of neither unknown prevalence nor incidence hence the number of infected patients showing symptoms are few [5]. Remarkably, several classes of parasites occur in allograft recipients, however the number of pathogenic parasites that could infect transplant recipients is about 5% and still does not reflect the actual incidence of the parasitic infections, however it is only for those causing transplanted organ to be rejected [6–8].

Importantly, parasitic infections could be considered as a major cause of distressing and death after transplantation [9]. Surprisingly, the incidence of first infections in the earliest 3 years ensuing kidney transplantation is 45.0 per 100 patient-years of follow-up [10]. Oddly, emerging intestinal parasites have become a significant opportunistic pathogens accountable for the most clinically important infections in immune-compromised patients especially renal transplant patients [11, 12]. Although parasites might be occupied through the feco-oral route, it is also might be reactivated in the immunocompromised host infected transplanted organ or being as dormant stages in the recipient itself [6], or by means of acquired transmission occurred by transplanted organ into a naive recipient [1]. Intestinal parasitic infections among renal transplant recipients requires careful deliberation as the infection may exacerbate with difficulties upon treatment [5]. Immunosuppressive drugs used by renal transplant recipient were reported to increase the susceptibility of harboring parasitic infection [11]. Several studies discussed presence of certain parasites detected in immunocompromised patients specially transplant recipients [11, 13–15].

Remarkably, opportunistic parasitic infections occurring in renal allograft recipients are well-known agents not only for causing diarrhea, but also are most important public health problem in developing countries [16]. Furthermore, intestinal parasitosis has been documented as a clinically significant infection not merely in immunocompromised patients, but also in hemodialysis [17–19].

In Sudan, intestinal parasites were transmitted via contaminated fresh vegetables that eaten on daily basis [20], indicating that public health being at high risk of infection with intestinal parasites, resulting in the increase of parasitic diseases harboring by susceptible immunocompromised patients particularly those of renal transplantation. Nevertheless, to our knowledge there have been very scarce studies addressing this issue in Sudan [21]. The aim of this study is to determine the frequency of enteroparasitosis among renal transplant recipients compared to healthy individuals in Khartoum state, Sudan.

## Main text

### Materials and methods

A case–control hospital-based study carried out in a period between November 2012 and May 2013; 300 renal transplant recipients were enrolled in the study while they attending Sudanese Kidney Association hospital in Khartoum state, Sudan. Another 300 normal individuals were recruited from the general population; taking into consideration the absence of any intestinal symptoms. Matching of cases and controls in terms of socio-economic and living condition status has been done through questionnaire survey for the recruited controls, and to ensure that the subjects chosen to participate as healthy have not undergone renal transplantation in the past. Stool specimens were collected in sterile, screw capped disposable plastic containers, and immediately transported to the Department of Microbiology—University of Khartoum, for parasitological studies. Additionally, dialysis duration and previous usage of immunosuppressive medications or any other medications gleaned were recorded using a well-designed questionnaire. Direct smears were applied to the patients’ and the healthy participants’ samples on a clean slide using a wooden stick, then immediately two drops of normal saline were added, and gently mixed with the specimen. Then the specimens were covered by coverslip and examined using a low power objective (×10) and (×40) for identification of parasites. Moreover, formalin-ethyl acetate concentration technique was performed. In this context the sediments were examined for intestinal protozoa, eggs and larvae of intestinal helminths using light microscope [22]. Another smear was prepared and stained by modified Ziehl–Neelsen acid-fast stain for the detection of *Cryptosporidium* oocyst according to guidelines by Casemore et al. [23].

#### Statistical analysis

Data analysis was performed using the Statistical Package for Social Sciences (SPSS) v20.0. Chi square test was used to find out the association between parasitic infections among renal transplant and healthy group. Additionally, odds ratio for both case and control groups for harboring intestinal parasites were also been calculated.

### Results

#### Study characteristics

A total of 600 fecal samples were collected during this study. 300 were renal transplant recipients their ages ranged between 11 and 80 years with a mean age of 43 ± 13.4 years. Of these, 242 (80.7%) were males mean age 43 ± 11.28 and 58 (19.3%) were females mean age 41 ± 13.41, while the healthy participants were 300 individuals, their age ranges between 9 and 75 years with a mean age of 44 ± 13.1 years. Of these 255 (85%) were males mean age 50 ± 19.18, 48 (16%) were females mean age 43 ± 13.84. Most of the study participants were categorized as middle income (68.6%) 206 were cases and 206 controls, while (31.4%) were of low income condition; 100 were cases and 88 were controls. No significant found for the association of socio-economic status with the prevalence of intestinal parasitosis, P value 0.503.

Family history of renal failure was reported among 39 (13.0%) of renal transplant recipients, while none of the participated controls had any family history of renal failure, P value 0.000. Also, all transplanted recipients assigned for dialysis before transplantation process, their dialysis periods were vary; 193 (64.4%) assigned for less than 3 years, 74 (24.6%) from 4 to 7 years, 22 (7.3%) from 8 to 12 years, 8 (2.7%) from 13 to 16 years and for more than 16 years there was only one recipient.

All renal transplant recipients were using several combinations of treatment; Cyclosporine with Cortisone were 62 (20.7%), Cyclosporine with Azathioprine were 116 (38.6%), Tacrolimus combined with Mycophenolate mofetil were 83 (27.7%) and 39 (13.0%) were using Cyclosporine combined with Cortisone and Mycophenolate mofetil.

Anti-parasitic medications used by the renal transplant recipients and the healthy controls were vary depending on the causative agent of the enteroparasitosis; mostly intestinal protozoa was treated using Metronidazole tabs taking into consideration the increase in blood level of cyclosporine, therefore kidney function monitoring was done while taking the course of medication. While all infections caused by helminthes parasites were treated with Praziquantel, and infections caused by the roundworms were treated with Mebendazole. Nevertheless, cryptosporidium infections were treated by Nitazoxanide.

#### Enteroparasitosis prevalence

A total of 118/600 (19.7%) stool samples were found to be positive for intestinal parasitic infection, 64/300 (21.3%) belong to renal transplant recipients and 54/300 (18.0%) to the considered healthy participants. Odds ratio for harboring intestinal parasites among the case and controls was 1.24 (CI 95%, 0.83–1.86). Interestingly, of both renal transplant and healthy participants, the most detected parasites were *Entamoeba histolytica/dispar* with frequency of 24 (37.5%) and 21 (38.9%) respectively. No significant difference in presence of *E. histolytica/dispar* infections between cases and controls (P > 0.05). The frequency of other detected intestinal parasites in renal transplant recipients include *Entamoeba coli* 21 (32.8%), *Giardia lambelia* 9 (14.1%), *Cryptosporidium parvum* 5 (7.81%), *Ascaris lumbricoides* 2 (3.12%), *Enterobius vermicularis* 1 (1.5%), *Strongyloides stercoralis* 1 (1.5%), and *Hymenolepis nana* 1 (1.5%). Concerning control group, the most detected parasites among the 54 positive stool samples were *E. coli* 18 (33.3%), *G. lambelia* were (18.51%), *C. parvum* (3.7%), *A. lumbricoides* (3.7%), and *E. vermicularis* (1.85%). Across the total studied participants there were no significant differences between transplanted recipients and control groups in the frequency distribution of intestinal parasites infecting both groups (Table 1).

**Table 1:** Illustrate the intestinal parasites distribution across the study population

Parasites detected	Transplant recipients	Healthy participants	Total	P value
Negative	236 (78.7%)	246 (82.0%)	482 (80.3%)	NA
*Entamoeba histolytica/dispar*	24 (8.0%)	21 (7.0%)	45 (7.5%)	0.756
*Entamoeba coli*	21 (7.0%)	18 (6.0%)	39 (6.5%)	0.740
*Cryptosporidium parvum*	5 (1.7%)	2 (0.7%)	7 (1.2%)	0.450
*Giardia lambelia*	9 (3.0%)	10 (3.3%)	19 (3.2%)	1.000
*Enterobius vermicularis*	1 (0.3%)	1 (0.3%)	2 (0.3%)	1.000
*Hymenolepis nana*	1 (0.3%)	0	1 (0.2%)	1.000
*Ascaris lumbricoides*	2 (0.7%)	2 (0.7%)	4 (0.7%)	1.000
*Strongyloides stercoralis*	1 (0.3%)	0	1 (0.2%)	1.000
Total	300 (50.0%)	300 (50.0%)	600 (100%)	

**Fig. 1:**
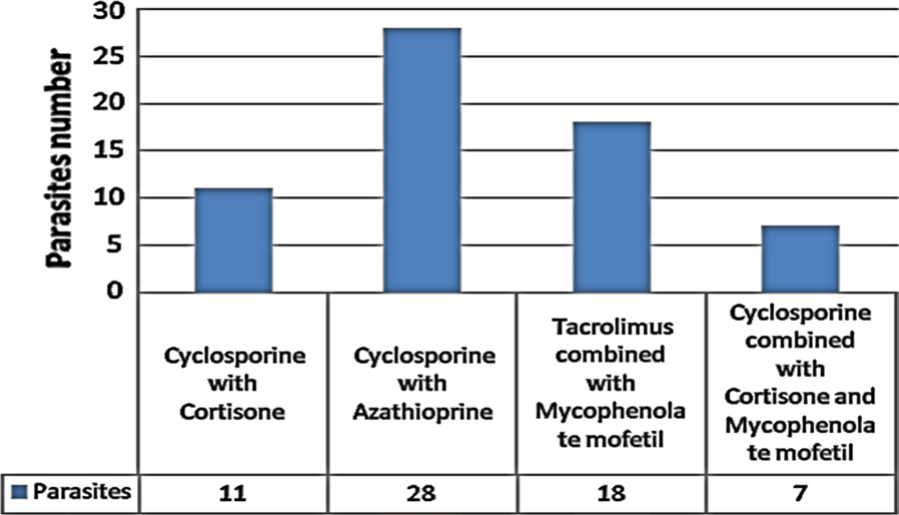
The frequency of intestinal parasitosis among the different regimens groups used for the treatment of the renal transplanted recipients

Concerning the infected renal transplant recipient, intestinal parasitosis was more prevalent among recipient taking Cyclosporine with Azathioprine treatments 28 (43.8%), while recipients taking the combination of Cyclosporine combined with Cortisone and Mycophenolate mofetil were the least group showing intestinal infection; 7 (10.9%) (Fig. 1).

The illustration of intestinal parasites infecting the renal transplant recipient, *E. histolytica* was found to be the most prevalent parasite followed by *E. coli*; 24 (37.5%), 21 (32.8%) respectively. The frequencies of the other parasites were shown in Table 2.

**Table 2:** Frequency of intestinal parasites among the different regimens used with renal transplant recipients

Treatment	E. H/D	E. C	C. P	G. L	E. V	H. N	A. L	S. S	Total
Cyclosporine with Cortisone	3 (27.3%)	5 (45.5%)	0 (0.0%)	2 (18.2%)	0 (0.0%)	0 (0.0%)	1 (9.1%)	0 (0.0%)	11 (17.2%)
Cyclosporine with Azathioprine	11 (39.3%)	5 (17.9%)	3 (10.7%)	6 (21.4%)	1 (3.6%)	1 (3.6%)	0 (0.0%)	1 (3.6%)	28 (43.8%)
Tacrolimus combined with Mycophenolate mofetil	8 (44.4%)	10 (55.6%)	0 (0.0%)	0 (0.0%)	0 (0.0%)	0 (0.0%)	0 (0.0%)	0 (0.0%)	18 (28.1%)
Cyclosporine combined with Cortisone and Mycophenolate mofetil	2 (28.6%)	1 (14.3%)	2 (28.6%)	1 (14.3%)	0 (0.0%)	0 (0.0%)	1 (14.3%)	0 (0.0%)	7 (10.9%)
Total	24 (37.5%)	21 (32.8%)	5 (7.8%)	9 (14.0%)	1 (1.6%)	1 (1.6%)	2 (3.1%)	1 (1.6%)	64 (100%)

E. H/D: *Entamoeba histolytica/dispare*, E. C: *Entamoeba coli*, C. P: *Cryptosporidium parvum*, G. L: *Giardia lambelia*, E. V: *Enterobius vermicularis*, H. N: *Hymenolepis nana*, A. L: *Ascaris lumbricoides*, S. S: *Strongyloides stercoralis*

### Discussion

Frequency of intestinal parasites in renal transplant recipients is not well-known in Sudan, so we had to compare our results to other studies carried out on renal transplant recipients worldwide. In the present study, *E. histolytica/dispare* was the most prevalent parasite detected in both groups, followed by *E. coli* and *G. lambelia*. Our results agree with previous reported results [12]. Comparing to previous reports on the prevalence of intestinal parasites in developing countries, this results were also in agreement [24, 25]. Also in industrial countries such as Albania [26], Poland [27, 28] and Turkey [29]. However, the higher rates in these communities attributed to improper hygiene and agricultural backgrounds.

*Cryptosporidium* infection is prevalent in communities with overcrowding and low level sanitation [14], and its prevalence reaches up to 36% in certain developing countries [30]. Given worldwide distribution and can be transmitted by contaminated food and water [20, 31], our results are in-discordant with Udgiri et al. [16], reported the incidence of *C. parvum* infections in India. They pointed 12 out of 60 patients had *Cryptosporidium* oocysts., also in Saudi Arabia *C. parvum* were reported to be the most prevalent intestinal parasite [31]. However, In Sudan the exact coccidian infection rates are still not known.

The prevalence of *H. nana* was found to be (0.3%), this finding was in accordance with growing study also with previous reports from Albania [26]. Whereas, only one case of *A. lumbricoides* has been diagnosed in this study albeit there are mounting evidence of studies pertaining to Ascariasis not only among immunocompromised hosts [4] but also across the population of different regions [26–28, 32–34].

The data presented in this study highlighted the distribution of *S. stercoralis* among renal transplant recipients; showing only one case has been diagnosed. Notwithstanding, in emerging body of study, *S. stercoralis* was reported as the most prevalent parasite, and it can cause an overwhelming disease in transplant recipients [9] with severe type of infection in non immunocompromised patients as reported in France [35] and Spain [36, 37].

Although the recipient was taking Cyclosporine A which known to become a cornerstone in prophylactic immunosuppression. Hopefully, the use of cyclosporine has lessened the incidence of strongyloidiasis in renal transplant recipients [38, 39]. None surprisingly, high-dose of corticosteroid among infected donors can increase rates and intensity of *S. stercoralis* transmission [40].

The data presented in the current study found no conclusive data of difference among transplanted subjects compared to non-transplanted healthy population for harboring parasitic infections. The explanation might lie in the fact that these parasitic infections are normally distributed in the community and transmitted through contaminated food and water resources [20]. This findings may agree with several studies stating that intestinal parasitic infections in immune-compromised patients depend mainly on the frequency of intestinal parasitism in the local community, showing no correlation to immune status of the patients [13, 41]. Also this finding is similar to results attained from HIV patients in comparison with healthy individuals [13, 42, 43] and in parasitic infections among renal transplanted patients in comparison with non-transplanted controls in Iran [44].

### Conclusions

Examination of a stool sample will benefit all patients on long-lasting immunosuppressive therapy to prevent the disease’s distressing and death and improve their quality of life.

## Limitations

- Presence of the parasitic infection among renal transplanted recipients still unknown whether the exposure occurred before or after the transplantation therefore examining transplant patients before and after the transplant and at different time intervals following transplantation would significantly improve future studies.
- A need for accurate estimate for the parasitic infections across the population is required.
